# Numerical Algorithm for Delta of Asian Option

**DOI:** 10.1155/2015/692847

**Published:** 2015-07-09

**Authors:** Boxiang Zhang, Yang Yu, Weiguo Wang

**Affiliations:** School of Economics, Dongbei University of Finance and Economics, 217 Jianshan Street, Dalian, Liaoning 116023, China

## Abstract

We study the numerical solution of the Greeks of Asian options. In particular, we derive a close form solution of Δ of Asian geometric option and use this analytical form as a control to numerically calculate Δ of Asian arithmetic option, which is known to have no explicit close form solution. We implement our proposed numerical method and compare the standard error with other classical variance reduction methods. Our method provides an efficient solution to the hedging strategy with Asian options.

## 1. Introduction

Asian options are referred to as securities with payoffs that depend on the average of the underlying stock price over a time interval. It got its name around 1987, when David Spaughton and Mark Standish worked for Bankers Trust in Tokyo, where they developed the first commercially used pricing formula for options linked to the average price of crude oil. They called the options “Asian options” since they were in Asia (see Falloon and Turner [1]). Asian options have appealing features that attract many investors. For example, end-users of energies or commodities tend to be exposed to the average prices over time, so Asian options suit their needs. Asian options are also popular among international corporations, who have ongoing currency exposures. Asian options tend to be less expensive than comparable Vanilla options, since the volatility in the average value of the underlying asset tends to be less than its spot value. Asian options also reduce the risk of price manipulation of underlying asset that is thinly traded.

The payoff of Asian arithmetic average call option with strike price *K* is given by

(1) $$\begin{array}{c} \Phi \left(\;S\;\right)=\max\left\{\;\frac{1}{T}\overset{T}{\underset{0}{\int }}S_{t}dt-K,0\;\right\}. \end{array}$$

Since no analytical solution is known, a variety of numerical approximation techniques have been developed to analyze the Asian arithmetic average option. Many authors are devoted to the numerical approximation of the close form formula (see Turnbull and Wakeman [2], Vorst [3], Levy [4], and Levy and Turnbull [5]). Monte Carlo simulation could be a nice approach (see Broadie and Glasserman [6] and Kemna and Vorst [7]), but it can be computational expensive without variation reduction method. It should also be noted that the discretization of the continuous process could introduce errors (see Broadie et al. [8]). Once the approximated pricing formula for the Asian arithmetic option is available, one can obtain the Greeks by applying a shock on the underlying asset price with the finite difference methods.

In this paper, we study the Greeks of Asian arithmetic call option. In particular, we will implement a numerical scheme to compute Δ of Asian arithmetic call option, by Monte Carlo method with a control variate. In Section 3, we briefly introduce the general principle of Monte Carlo method with some variance reduction techniques. In Section 4, we derive a close form pricing formula for the Asian geometric average call option. As a consequence, we obtain an analytical formula for Δ of Asian geometric average call option, which will be used as a control variate in the Monte Carlo simulation. In the last section, we describe the numerical scheme to compute Δ of Asian arithmetic average call option and compare our results with other variance reduction techniques.

## 2. Existing Literature

The Monte Carlo method can be used to price a wide range of exotic options as well as to analyze their Greeks, especially when the close form solutions do not exist. However, due to the reason of biased estimation and high computation cost, many modified Monte Carlo methods were proposed to estimate the Greeks by simulation.

Broadie and Glasserman [6] developed a method called infinitesimal perturbation analysis, which is based on the relationship between the payoffs and the Greeks of interest. Unlike the infinitesimal perturbation analysis, the likelihood ratio method is based on the probability density function of underlying price and the Greeks. Both methods mentioned above provide unbiased estimators but differ in applicability and effectiveness. Fournié et al. [9] suggested a framework for Greeks estimating that they showed that, under some certain circumstance, the Greeks can be represented by the product of the option payoff and a weight function, which is given by Malliavin calculus theory (see Kohatsu-Higa and Montero [10]).

In order to reduce the variance of the estimators, many techniques have been introduced. The most effective variance reduction technique is the control variate method. In the case of Asian option, the payoff of geometric Asian option is set to be a control variate in order to improve the effectiveness of the payoffs of algorithm Asian option prices.

Other methods include analytic method and finite difference approach. See Boyle and Potapchik [11] for an extensive survey of relevant literature.

## 3. The Monte Carlo Framework and Variance Reduction Techniques

Nowadays the advance of financial engineering has introduced lots of demands on using Monte Carlo simulations to price the options. Monte Carlo methods are important in many situations where the option price admits a simple risk-neutral valuation formula but not a tractable PDE formulation, like Asian option, for example. As a consequence, the Greeks associate with these options do not admit close form formula but can be obtained numerically by a combination of finite difference method and Monte Carlo method. Let us first outline some general principle of Monte Carlo method and variance reduction techniques.

### 3.1. General Principle of Monte Carlo Method

Let *X* be random variable and let *g*(*x*) be measurable function such that *μ*_*X*_ = *𝔼g*(*X*) with *𝔼*(|*g*(*X*)|) < *∞*. Then we can numerically simulate *n* independent replicas of *X*, denoted by *X*_1_,…, *X*_*n*_, and approximate *μ*_*X*_ by the Monte Carlo estimator:

(2) $$\begin{array}{c} {\hat{\mu }}_{X}^{n}=\frac{1}{n}\overset{n}{\underset{k=1}{\sum }}g\left(\;X_{k}\;\right). \end{array}$$

By the law of large number, we know that ${\hat{\mu }}_{X}^{n}$ is a good estimate of *μ*_*X*_ in the sense that

(3) $$\begin{array}{c} {\hat{\mu }}_{X}^{n}⟶\mu_{X},\;\text{a.s. as }n⟶\infty . \end{array}$$

However, it is important to understand that Monte Carlo method is never exact. The estimating error can be quantified by the so-called standard error defined by

(4) $$\begin{array}{c} s=\sqrt{\frac{1}{n-1}\overset{n}{\underset{i=1}{\sum }}{\left(g\left(X_{i}\right)-{\hat{\mu }}_{X}^{n}\right)}^{2}}. \end{array}$$

If the standard deviation is small, then it is a good sign that with high probability our Monte Carlo result would be close to the true value. Otherwise, the high standard deviation indicates that our result might be deviating from the true value. It is also important to observe that the standard deviation follows square root rule, which suggests that the convergence is relatively slow. As a consequence, if the standard deviation is high, obtaining a promising accuracy would require high computational cost. Next, we briefly introduce some classical variance reduction techniques.

### 3.2. Common Random Number Method

The Common Random Number (CRN for short) method is one of the classic variance reduction techniques. The main idea is to use the same random number sequence when the target is the difference of two random variables which depends on the underlying random number sequence. For simplicity, suppose now that we want to estimate *μ*_*X*_ = *𝔼*(*X*) = *𝔼*(*X*_1_ − *X*_2_), where *X*_1_ and *X*_2_ are two random variables. It is obvious to see that

(5) $$\begin{array}{c} \mathrm{var}\left(\;X\;\right)=\mathrm{var}\left(\;X_{1}\;\right)+\mathrm{var}\left(\;X_{2}\;\right)-2\mathrm{cov}\left(\;X_{1},X_{2}\;\right). \end{array}$$

If *X*_1_ and *X*_2_ are positively correlated, then we could reduce the variance of the estimator. Now suppose *X*_1_ = *g*_1_(*Z*) and *X*_2_ = *g*_2_(*Z*), where *Z* is a standard normal random vector and *g*_1_(*x*) and *g*_2_(*x*) have the same monotonicity. Then the CRN estimator is defined by the following:

(6) $$\begin{array}{c} {\hat{\mu }}_{\text{CRN}}^{n}=\frac{1}{n}\overset{n}{\underset{i=1}{\sum }}\left(\;g_{1}\left(\;Z_{i}\;\right)-g_{2}\left(\;Z_{i}\;\right)\;\right), \end{array}$$

where *Z*_*i*_'s are iid normal distributed. It is easily seen that the variance of ${\hat{\mu }}_{CRN}^{n}$ is less than the crude Monte Carlo estimator ${\hat{\mu }}_{X}$.

### 3.3. Control Variate Method

Now let us briefly introduce the general idea of control variate method. We want to estimate *μ*_*X*_∶ = *𝔼*(*X*). Now if we can find another random variable *Y* with known mean *𝔼*(*Y*), then we can construct a family of unbiased estimators of *μ*_*X*_:

(7) $$\begin{array}{c} {\hat{\mu }}_{C,X}^{n}={\hat{\mu }}_{X}^{n}+\beta \left(\;Y-E\left(\;Y\;\right)\;\right), \end{array}$$

where

(8) $$\begin{array}{c} {\hat{\mu }}_{X}^{n}=\frac{1}{n}\overset{n}{\underset{k=1}{\sum }}X_{k}. \end{array}$$

From the very definition, it is easily seen that ${\hat{\mu }}_{C,X}^{n}$ are unbiased estimators. Indeed,

(9) $$\begin{array}{c} E\left(\;{\hat{\mu }}_{C,X}^{n}\;\right)=E{\hat{\mu }}_{X}^{n}+\beta \left(\;E\left(\;Y\;\right)-E\left(\;Y\;\right)\;\right)=\mu_{X}. \end{array}$$

Moreover, we have

(10) $$\begin{array}{c} \mathrm{var}\left(\;{\hat{\mu }}_{C,X}^{n}\;\right)=\mathrm{var}\left(\;{\hat{\mu }}_{X}^{n}\;\right)+2\beta \mathrm{cov}\left(\;{\hat{\mu }}_{X}^{n},Y\;\right)+\beta^{2}\mathrm{var}\left(\;Y\;\right). \end{array}$$

To minimize the variance of ${\hat{\mu }}_{C,X}^{n}$, we could choose

(11) $$\begin{array}{c} \beta_{\min}=-\frac{\mathrm{cov}\left({\hat{\mu }}_{X}^{n},Y\right)}{\mathrm{var}\left(Y\right)}. \end{array}$$

As a consequence, to construct a good estimator, we need to choose a random variable *Y* that is positively correlated to *X*. Usually, if we could find a random variable *Y* with known mean *𝔼*(*Y*) that is positively correlated to *X*, we could simply choose *β* = −1. Intuitively, we could think of *θ*_*e*_ = (*Y* − *𝔼*(*Y*)) as an error adjustment to the unadjusted estimator ${\hat{\mu }}_{X}^{n}$. If the standard error is high, then deviation of ${\hat{\mu }}_{X}^{n}$ from *𝔼*(*x*) is large. At the same time, *θ*_*e*_ is large, by the fact that *X* and *Y* are positively correlated. It would reduce the standard error of ${\hat{\mu }}_{C,X}^{n}$, even with a moderate size of *n*.

## 4. Asian Geometric Option as a Control Variate

The payoff of Asian geometric option is given by

(12) $$\begin{array}{c} \Phi \left(\;S\;\right)=\max\left\{\;e^{\left(1/T\right)\int_{0}^{T}\log S\left(t\right)dt}-K,0\;\right\}. \end{array}$$

Under the classical Black-Scholes model, we know that the underlying asset *S*(*t*) is a geometric Brownian motion given by (see Bjork [12] for more details)

(13) $$\begin{array}{c} S\left(\;t\;\right)=S_{0}e^{\left(r-\sigma^{2}/2\right)t+\sigma W_{t}}. \end{array}$$

**Proposition 1 .**

Let *C*_*geo*_ be the price of Asian geometric call option under Black-Scholes model. Then

(14) Cgeo=S0e−r+σ2/6T/2Nd1−Ke−rTNd2,Δgeo=∂Cgeo∂S=e−r+σ2/6T/2Nd1+1σ2πT/3e−rT/2+Tσ2/12+d12/2−KSe−rT+d22/2,

where

(15) d1=log⁡S0/K+T/2r+σ2/6σT/3,d2=log⁡S0/K+T/2r−σ2/2σT/3.

**Proof**

It is important to note that the average price *e* ^(1/*T*)∫_0_^*T*^log*S*(*t*)*dt*^ follows log normal distribution. Indeed, we have

(16) e1/T∫0Tlog⁡Stdte1/T∫0Tlog⁡S0+r−σ2/2t+σWtdt=elog⁡S0+T/2r−σ2/2+σ/T∫0TWtdt=S0eT/2r−σ2/2+σ/T∫0TWtdt.

Observe that the integral ∫_0_^*T*^ *W*_*t*_ *dt* is normal with mean 0 and variance

(17) E∫0TWtdt2E∫0T∫0TWsWtds dt=∫0T∫0TEWsWtds dt=∫0T∫0Ts∧tds dt=2∫0T∫0ts ds dt=T33.

To simplify our notation, we denote *Z* = (*T*/2)(*r* − *σ* ^2^/2) + (*σ*/*T*)∫_0_^*T*^ *W*_*t*_ *dt*. By the argument above, we know that $Z\sim N(T/2(r-\sigma^{2}/2),\sigma \sqrt{T/3})$. Now by the risk-neutral valuation formula, we have

(18) Cgeoe−rTEQS0eZ−K+ ∣ F0=e−rTEQS0eZ−K1S0eZ>K=e−rTEQS0eZ1S0eZ>K−Ke−rTPS0eZ>K.

On the one hand, we have

(19) PS0eZ>K=PZ>log⁡KS0=PZ−T/2r−σ2/2σT/3>log⁡K/S0−T/2r−σ2/2σT/3=Nd2,

where we used the fact that $\left(Z-\left(T/2\right)(r-\sigma^{2}/2)\right)/\sigma \sqrt{T/3}$ follows standard normal distribution and

(20) $$\begin{array}{c} d_{2}=\frac{\log\left(S_{0}/K\right)+\left(T/2\right)\left(r-\sigma^{2}/2\right)}{\sigma \sqrt{T/3}}. \end{array}$$

On the other hand, we got 
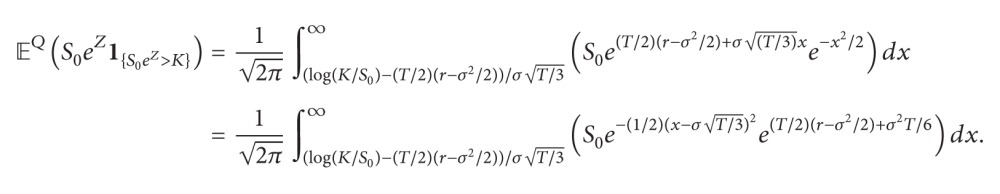
(21)
By change of variable $u=x-\sigma \sqrt{T/3}$ we have
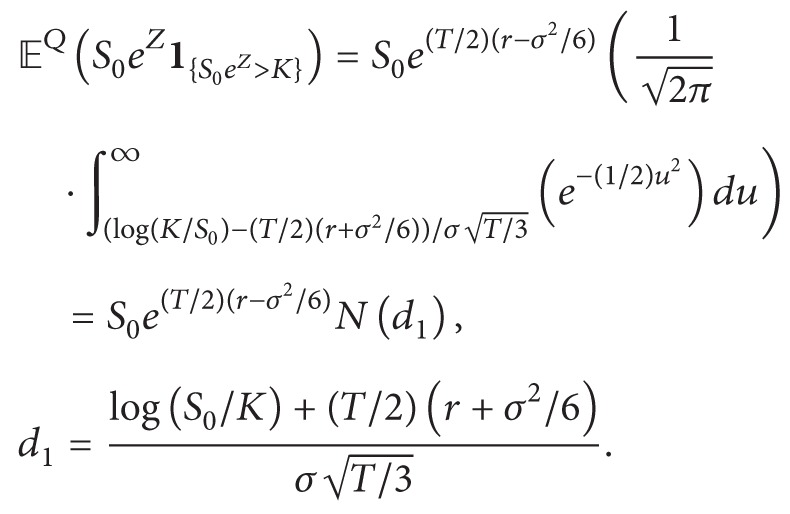
(22)Now putting all pieces together, we have

(23) $$\begin{array}{c} C_{\text{geo}}=S_{0}e^{-\left(r+\sigma^{2}/6\right)\left(T/2\right)}N\left(\;d_{1}\;\right)-Ke^{-rT}N\left(\;d_{2}\;\right). \end{array}$$

Now we are ready to compute Δ_geo_. By chain rule, we have

(24) ∂Cgeo∂S=e−r+σ2/6T/2Nd1+Se−r+σ2/6T/2φd1∂d1∂S−Ke−rTφd2∂d2∂S=e−r+σ2/6T/2Nd1+Se−r+σ2/6T/2φd1−Ke−rTφd21SσT/3=e−r+σ2/6T/2Nd1+1σ2πT/3e−rT/2+Tσ2/12+d12/2−KSe−rT+d22/2.

## 5. Numerical Computations

In this part, we describe the numerical scheme of the computation of Asian arithmetic option Greeks with control variate method. Let us first remind the reader of the model. Under the risk-neutral probability measure, the dynamics of the underlying asset *S*_*t*_ is described by the following stochastic differential equation:

(25) $$\begin{array}{c} S_{t}=S_{0}+\overset{t}{\underset{0}{\int }}rS_{u}du+\overset{t}{\underset{0}{\int }}\sigma S_{u}dW_{u}. \end{array}$$

The solution is given by the geometric Brownian motion

(26) $$\begin{array}{c} S_{t}=S_{0}e^{\left(r-\sigma^{2}/2\right)t+\sigma W_{t}}. \end{array}$$

The payoff of Asian arithmetic option is given by

(27) $$\begin{array}{c} \Phi \left(\;S\;\right)=\max\left\{\;\frac{1}{T}\overset{T}{\underset{0}{\int }}S_{t}dt-K,0\;\right\}. \end{array}$$

Then the numerical scheme follows the next several steps.

**Step 1 (sample path generation).**

Let us first fix some parameters: time to maturity *T* > 0, time steps *m* ∈ *ℕ*, initial price *S* > 0, and initial underlying price shock *ϵ* > 0. We denote *t*_*i*_ = (*T*/*m*)*i*, and then we generate sample paths *S*_*t*_*i*__ and ${\tilde{S}}_{t_{i}}$ by the following iterative steps:

(28) Sti=Sti−1er−σ2/2T/n+σT/nZi,S0=S,S~ti=S~ti−1er−σ2/2T/n+σT/nZi,S~0=S+ϵ,

where *Z*_1_,…, *Z*_*m*_ are *m* copies of iid normal random numbers. It is important to observe that we used the same random numbers for both paths *S*_*t*_ and ${\tilde{S}}_{t}$, for the purpose of variance reduction.

**Step 2 (approximating the payoff functions of both Asian arithmetic and Asian geometric option).**

We first use the Riemann sum to numerically approximate the integral. Indeed,

(29) $$\begin{array}{c} \frac{1}{T}\overset{T}{\underset{0}{\int }}S_{t}dt\approx \frac{1}{T}\overset{n}{\underset{i=1}{\sum }}S_{t_{i}}\frac{T}{n}=\frac{1}{n}\overset{n}{\underset{i=1}{\sum }}S_{t_{i}}. \end{array}$$

Next, we simulate *n* copies of the Asian arithmetic option payoff

(30) ΦAjS=max⁡1n∑i=1nStij−K,0j=1,…,m,Φ~AjS=max⁡1n∑i=1nS~tij−K,0j=1,…,m.

Similarly, we simulate *n* copies of the Asian geometric option payoff

(31) ΦGjS=max⁡e1/n∑i=1nlog⁡Sti−K,0j=1,…,m,Φ~GjS=max⁡e1/n∑i=1nlog⁡S~ti−K,0j=1,…,m.

**Step 3 (finite difference approximation).**

Let *C*_*A*_(*S*) be the Asian arithmetic option price under the Black-Scholes model, and then

(32) ∂CA∂S≈CAS+ϵ−CASϵ=e−rTEQΦ~AS−EQΦASϵ≈e−rT1/m∑j=1nΦ~AjS−1/m∑j=1nΦAjSϵ=e−rTmϵ∑j=1nΦ~AjS−ΦAjS≕Δ^A.

Similarly, we construct the sample estimator for Asian geometric option price *C*_*G*_(*S*),

(33) $$\begin{array}{c} \frac{\partial C_{G}}{\partial S}\approx \frac{e^{-rT}}{m\epsilon }\overset{n}{\underset{j=1}{\sum }}\left(\;{\tilde{\Phi }}_{G}^{\left(j\right)}\left(\;S\;\right)-\Phi_{G}^{\left(j\right)}\left(\;S\;\right)\;\right)≕{\hat{\Delta }}_{G}. \end{array}$$

**Step 4 (define the control sample estimator for Asian geometric option Greeks Δ).**

Consider

(34) $$\begin{array}{c} {\hat{\Delta }}_{\text{Control}}={\hat{\Delta }}_{A}-\left(\;{\hat{\Delta }}_{G}-\Delta_{G}\;\right). \end{array}$$

Next, let us take a look at the results of our proposed numerical simulation and compare it with the classic Common Random Number (CRN for short) with Table 1.

It is very clear that our proposed method is better than the CRN method in the sense that it introduce much smaller standard error than the classic CRN method with the same other parameters.

## 6. Conclusion

We study the numerical solution for the Delta of Asian arithmetic option, which was known to have no explicit analytical closed form solution. With the Delta of Asian geometric option as a control, we provided a simple, fast, intuitive, and reliable numerical solution in the sense that the standard errors of Monte Carlo simulation were reduced greatly. We also mention that, in recent years, many authors have been actively involved in the research of calculating Greeks of Asian type option using various approaches. For instance, in [13], the authors provided formulas for the Greeks of Asian arithmetic option, which involves the time integral of geometric Brownian motion. As stated in the paper, the results are consistent with the Monte Carlo simulation. In [14], with the advanced tool of Malliavin calculus, the authors give a quasiexplicit formula for the Asian option Greeks. Also, in [15], a PDE approach was utilized to understand the numerical value of the Asian option Greeks. To summarize, we believe that it is challenging but worth efforts to obtain more accurate value for the Asian arithmetic option Greeks, for the purpose of hedging strategy. Among various approximating methods, our numerical scheme provides a very neat and efficient choice when one wants to calculate hedge position of a portfolio that involves the Asian average options. Because the assumption of high correlation for the two variates needs to hold, the performance on other Greeks, however, still needs to be examined.

## Figures and Tables

**Table 1:** Monte Carlo simulation comparison table: common random number method versus control variate method for Asian arithmetic option.

*K*	*T*	Estimated Delta of Asian arithmetic option by CRN method	Standard error by CRN method	Estimated Delta of Asian arithmetic option by control variate method	Standard error by control variate method
50	1	0.8682	0.1971	0.8806	0.0239
	2	0.8140	0.1909	0.8351	0.0240
	3	0.7795	0.1868	0.7783	0.0298

60	1	0.5630	0.1588	0.5687	0.0199
	2	0.5875	0.1622	0.5466	0.0380
	3	0.6030	0.1643	0.5969	0.0278

70	1	0.2405	0.1038	0.2514	0.0283
	2	0.3668	0.1282	0.3569	0.0311
	3	0.4228	0.1376	0.3492	0.0249

The table above is based on the Asian arithmetic option with the following parameters: *S* = 60, *r* = 0.05, sigma = 0.3, sample size *n* = 5000, time step *m* = 500, and initial stock price shock epsilon = 0.1.
